# Genomic control of metastasis

**DOI:** 10.1038/s41416-020-01127-6

**Published:** 2020-11-04

**Authors:** Saroor A. Patel, Paulo Rodrigues, Ludovic Wesolowski, Sakari Vanharanta

**Affiliations:** MRC Cancer Unit, University of Cambridge, Hutchison/MRC Research Centre, Box 197, Cambridge Biomedical Campus, Cambridge, CB2 0XZ UK

**Keywords:** Metastasis, Cancer genetics

## Abstract

Metastasis remains the leading cause of cancer-associated mortality, and a detailed understanding of the metastatic process could suggest new therapeutic avenues. However, how metastatic phenotypes arise at the genomic level has remained a major open question in cancer biology. Comparative genetic studies of primary and metastatic cancers have revealed a complex picture of metastatic evolution with diverse temporal patterns and trajectories to dissemination. Whole-genome amplification is associated with metastatic cancer clones, but no metastasis-exclusive driver mutations have emerged. Instead, genetically activated oncogenic pathways that drive tumour initiation and early progression acquire metastatic traits by co-opting physiological programmes from stem cell, developmental and regenerative pathways. The functional consequences of oncogenic driver mutations therefore change via epigenetic mechanisms to promote metastasis. Increasing evidence is starting to uncover the molecular mechanisms that determine how specific oncogenic drivers interact with various physiological programmes, and what triggers their activation in support of metastasis. Detailed insight into the mechanisms that control metastasis is likely to reveal novel opportunities for intervention at different stages of metastatic progression.

## Background

Surgery can often cure primary cancers. However, considering that metastases account for the majority of cancer-associated patient fatalities, there is a clear unmet clinical need, and an understanding of how to optimally manage metastatic disease is still lacking for most cancer types. Despite the long history of metastasis research^1^ and the increase in our knowledge of the molecular mechanisms of metastasis gained, especially over the past couple of decades, the lack of effective therapies can at least partly be attributed to our poor understanding of the underlying biology. The formation of metastases is the result of a complex multistep cascade. In its simplest form, cells must disseminate from the primary tumour, either individually or by collective migration, enter the circulatory system (blood or lymphatic) as single cells or clusters, seed in a capillary bed or extravasate at a distant organ, survive in a foreign microenvironment and establish a secondary colony.^2–4^ Alternative scenarios can involve perineural invasion, metastasis via ascites or cerebrospinal fluid and intermediate metastases in the lymph nodes. Finally, disseminated cells may enter cellular dormancy, or their proliferation may be counterbalanced by death or elimination by the immune system, resulting in latent metastasis that may only become clinically detectable decades after primary tumour removal.^5^ The molecular mechanisms that enable cancer cells to progress through these various biological steps have been the focus of extensive investigation.^2,6,7^ For example, rearrangement of the actin cytoskeleton, modulation of the extracellular matrix (ECM) and the recruitment of certain immune cells facilitate efficient entry into the circulatory system.^8–10^ The formation of clusters of migratory cancer cells coated with platelets and metabolic reprogramming enable resistance to mechanical damage, innate immune response, anoikis and oxidative stress in the bloodstream.^11–16^ Arrest in a capillary bed and subsequent extravasation is influenced by factors that control cancer cell motility and endothelial cell disjunction.^6^ And, finally, the survival and establishment of secondary colonies depend upon a variety of factors that converge on key stem-cell support pathways, growth factor signalling, positional and mechanical pathways and inflammatory signalling.^5,17^

Despite the identification of a range of genes, molecules and pathways that contribute towards the successful completion of one or more of the metastatic steps, how the phenotypic traits that confer increased metastatic fitness are regulated remains largely unknown. Using a select set of examples of studies carried out over the past 15 years, in this review we will discuss the genetic and epigenetic determinants of metastasis and provide a perspective on the origin of metastatic phenotypes.

## The genetic landscape of metastasis

Given the complexity of metastasis, it is not surprising that colonisation of a secondary organ by tumour cells is highly inefficient.^18^ Accordingly, the number of cancer cells found in the circulation of patients greatly surpasses the number of detectable macroscopic lesions, and only half the number of patients with detectable disseminated tumour cells in their bone marrow go on to develop overt metastases in the long term.^19,20^ In mouse models, only 0.02% of melanoma cells injected into the portal vein went on to develop macro-metastases and even cell lines enriched for high metastatic potential suffer extensive losses during colonisation.^21–23^ The presence of distinct biological barriers and the inefficiency of cancer cells in overcoming them represent bottlenecks that can, at least in principle, result in the selection of specific mutations that promote metastasis. Indeed, extensive cancer genome resequencing efforts have explored the genetic origins of metastases^24–26^ and, although a clear genetic signature of metastasis is yet to emerge, these studies have provided unprecedented insight into the genetic patterns of metastatic evolution and mode of spread.

### The mode of spread and timing of metastasis

The trajectory of metastatic seeding has traditionally been viewed as an orderly multistep process from a founder cell in the primary tumour to distant organ colonisation. However, from detailed genetic mapping of the relationships between various regions in primary tumours and across metastatic sites in different cancer types, a far more complex—even chaotic—picture has emerged.

In colorectal cancer (CRC) some lymph node metastases are related to distant metastases, whereas others are not.^27^ Analyses of lymph node and distant organ metastases in CRC showed that these two types of metastases develop through different evolutionary mechanisms; lymph node metastases display a high level of interlesion heterogeneity in contrast to distant metastases, which were genetically similar to each other, suggesting that fewer primary tumour lineages are capable of seeding distant metastases than lymph node metastases.^28^ In melanoma, prostate and oesophageal cancers, distinct subclones spread directly from the primary tumour, each independently seeding multiple metastases.^29–32^ Similarly, in renal cancer and CRC, multiple metastases in different tissues can derive from a single subclone present in the primary tumour.^33,34^ However, in other cases of renal cancer, primary tumour clones at different stages of evolution seed metastases to different organs.^33^ In one patient with breast cancer, single-cell copy number variant (CNV) analysis of the primary tumour and its metastasis found that a single clonal expansion seeded the metastasis,^35^ whereas single-cell CNV and single-nucleotide variant (SNV) analyses in patients with CRC showed metastatic seeding by a single clone in one patient and multiple clones in another.^36^ These examples highlight the fact that some primary cancers contain multiple clones that are capable of forming metastases through different routes, whereas only one metastatic clone seems to exist in other cancers.

**Fig. 1:**
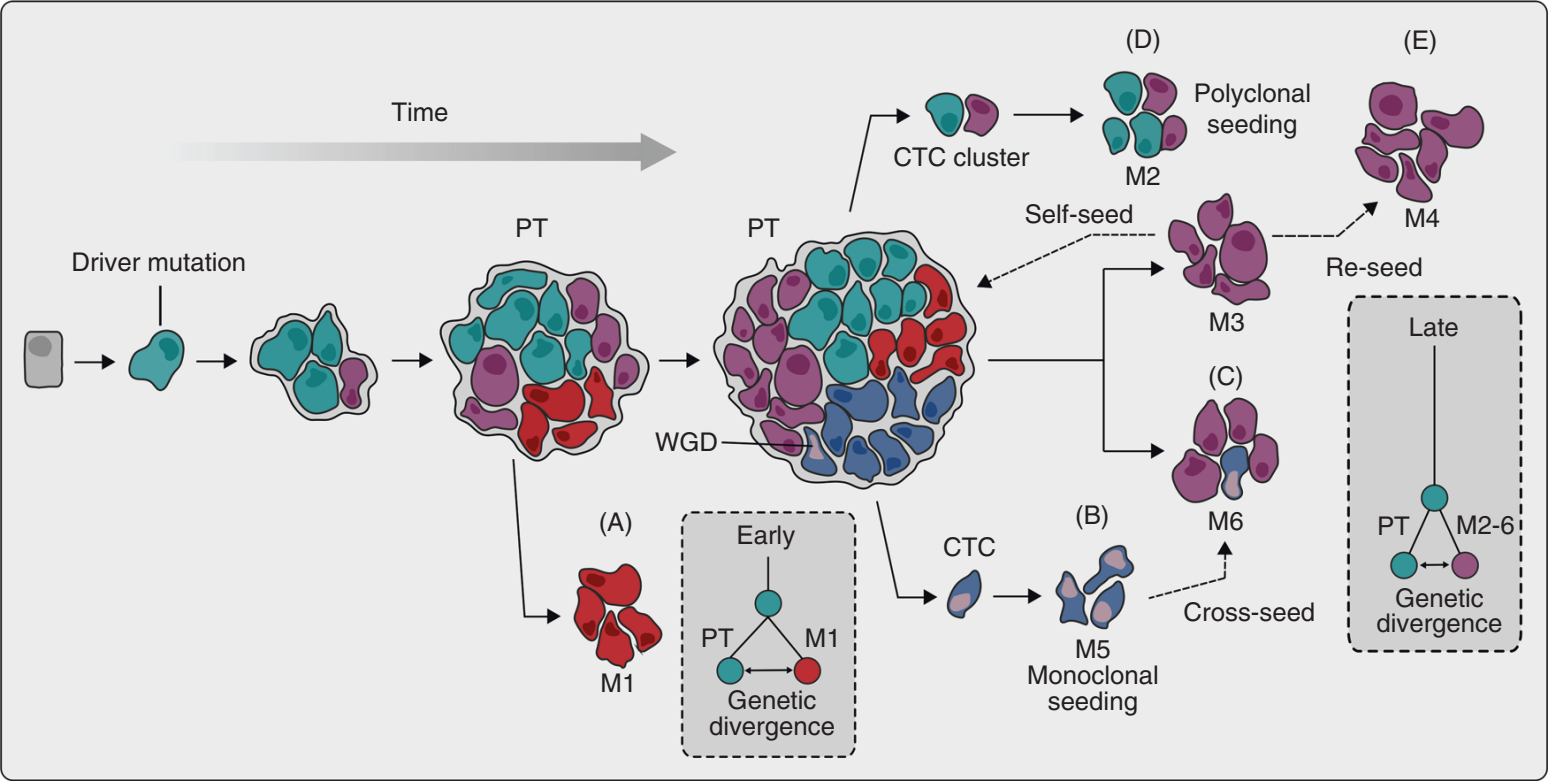
Complex temporal patterns and trajectories of metastatic dissemination. A cell acquires a set of driver mutations to form a primary tumour (PT). Aggressive primary tumour clones invade, circulate and seed secondary sites to form metastases (M) at various timepoints during tumour evolution. Clones leaving early have greater genetic divergence between the primary tumour and metastases (**a**) compared with  later disseminating clones (**c–e**). Clones can depart as single circulating tumour cells (CTC) or in clusters to seed monoclonal (**b**) and polyclonal (**d**) metastases, respectively. While driver mutations in metastases are the same as in the primary tumour, whole-genome doubling (WGD) is a more frequent genetic feature of metastases. Following seeding and outgrowth at a secondary site, metastatic clones can further disseminate to cross-seed (**c**), reseed (**e**) and self-seed, resulting in a complex clonal landscape.

Another level of complexity in the genetic origin of metastases is apparent when looking at individual metastases. In some cases, all the cells within a metastasis appear to be derived from a single clone (Fig. 1a, b),^37,38^ but individual metastases can contain multiple cancer clones in other cases (Fig. 1c, d).^29^ Multiple clones within a single metastasis can result from metastasis-to-metastasis seeding, a process known as cross-seeding, that can lead to highly complex patterns of tumour spread (Fig. 1c).^29,32^ In addition, clones from a metastasis can reseed to form a secondary metastasis (Fig. 1e).^32^ Furthermore, circulating tumour cell clusters have been shown to be highly metastatic and give rise to polyclonal metastasis (Fig. 1d).^11,39,40^ As deeper and more robust sequencing technologies emerge, studying genetic differences at a single-cell level will likely reveal even further complexities in the patterns of metastatic spread.

In addition to complex seeding patterns, the timing of clinically detectable metastatic progression varies across different metastases according to the type of primary cancer and the site of secondary disease. In pancreatic cancer, the development of a primary tumour typically takes around 12 years, but progression from the birth of the clone that seeds the primary to the seeding of the metastasis is comparatively fast, taking an average of 6.8 years.^41^ Data from renal cancer patients suggest that, depending on the secondary site, metastases manifest clinically at different timepoints, with a median of 15 years for pancreatic metastases but only 3 years for all other sites when compared with initial diagnosis.^33^ This variability could be a reflection of the timing of metastatic seeding as clones seeding metastasis to the pancreas had genetic lesions that were associated with less aggressive phenotypes. However, this difference could also be due, at least in part, to different modalities and effectiveness of clinical detection at particular sites. Late dissemination and seeding of the metastatic clone has been suggested by data gathered from breast cancer metastases (Fig. 1b–e).^42^ Likewise, advanced metastases from pancreatic and colon cancers are often genetically similar to their corresponding primary cancers.^37,38^ However, Hu et al. presented data from CRC, breast and lung cancer patients that suggest metastatic seeding might occur early, even before the primary tumour is clinically detectable, irrespective of the site of metastatic colonisation (Fig. 1a).^34,43^ Spatiotemporal data examining the timing and speed of spread from oesophageal cancer patients showed that in the majority of cases, metastatic spread from the primary site was rapid,^31^ similarly challenging the idea of metastasis as a late event. Interpretation of the genetic data are, however, complicated by the fact that the genetic divergence between the most advanced primary tumour clone and metastases, or the time at which a metastasis is clinically detected, does not necessarily reflect the timing of metastatic seeding.

Depending on the timing of metastatic seeding, the extent to which the primary tumour and established metastases evolve will vary. This parallel evolution leads to genetic differences, but it can also converge on shared genetic pathways to ultimately bring out the same consequence. Multi-region profiling of primary-metastatic prostate cancer pairs revealed in one case that metastases and the primary tumour were highly unrelated, suggestive of early divergence followed by parallel evolution, while, in another case, the metastatic subclone was found in the primary tumour but had accumulated additional mutations.^32^ Similar results have been described in renal cancer, where parallel evolution of metastatic clones leads to similar but not identical genetic alterations across different metastatic clones.^33,44^ In pancreatic cancer, analysis of the primary tumours and metastases from various distant sites revealed shared structural rearrangements that occurred early in tumour evolution and varying degrees of genetic divergence between primary and metastases, which was consistent with on-going parallel evolution.^45^

Based on the current data, it therefore appears that cancer types or even individual tumours are not constrained to a particular model of metastatic evolution. Rather, metastases are characterised by varied timings and trajectories of dissemination, in which distinct metastasis-competent clones leave the primary site at different timepoints to seed, reseed and cross-seed multiple metastases, all the while acquiring a more complex genetic landscape. The timing of seeding seems to be key in how these clones evolve. In some cancers, metastases are closely related to existing primary tumour clones, whereas in others, metastases have undergone significant further evolution in parallel.

### Mutational profiles of metastatic tumours

In general, specific driver gene mutations that are exclusive to metastases have not been identified to date despite extensive sequencing data from various cohorts and cancer types. In most cases, unique genetic alterations can be detected in the metastatic site when compared with their respective primary tumours but these changes are not consistent across metastases and also occur in primary sites in other contexts.^33,38,42,44,46,47^ In line with these observations, mathematical modelling using data from treatment-naïve patients predicted that the primary tumours and their metastases share the same driver mutations.^48^ Reiter at al. profiled 76 untreated metastases from 20 patients with various cancer types and found that all metastases within individual patients shared the same functional driver gene mutations. The authors did observe some inter-metastatic heterogeneity, but these heterogeneous mutations were predicted to be nonfunctional.^48^ These observations suggest that the mutational complements of the primary cell populations are sufficient for conferring metastatic capabilities.

Although metastases have similar mutational profiles to primary tumours, they appear to bear driver alterations at a higher frequency than primary tumours. Priestley et al. characterised 2520 samples of metastatic tumours from 22 solid cancer types.^24^ Consistent with other results, they found no evidence of driver mutations that were specific to metastases,^24^ but they did find alterations in the *MLK4* gene (which encodes mixed lineage kinase 4) frequently associated with metastatic tumours. *MLK4* upregulation has previously been linked to migratory and invasive phenotypes in breast cancer cells.^49^ They also identified candidate driver variants in over 98% of metastatic tumours; in 62% of patients, these variants were potentially therapeutically actionable.^24^ Similarly, another study profiled somatic alterations in 617 patients with metastatic breast cancer and concluded that the metastases were genetically more complex and had a higher mutational burden and clonal diversity when compared with early breast cancers.^50^ In addition, common germline variants of the *APOE* gene have been shown to be associated with different outcomes in melanoma. Mice expressing the human *APOE4* allele exhibited reduced metastasis relative to *APOE2* mice, suggesting certain genetic lesions can increase metastatic competence.^51^

Even though the same mutations seem to drive the growth of primary and metastatic cancers, the efficiency with which a particular clone seeds metastasis might still depend on its mutations. Turajilic et al. tested this notion directly by comparing the genetic architecture of different primary tumour clones with that of those that formed metastases in the same patients in renal cancer. The only genetic alteration that was significantly associated with metastasis was loss of 9p, a region which contains the cyclin-dependent kinase inhibitor 2A and 2B tumour suppressor loci. The authors also found that primary tumours with *PBRM1-SETD2* and *PBRM1-PI3K* driver mutations and high tumour heterogeneity were associated with attenuated progression.^33^ These results suggest that certain genetic alterations are capable of increasing metastatic competence but that these mutations are not exclusive to metastasis as they are already selected for in primary tumours.

Further evidence for the lack of a unifying metastasis-specific signature comes from experimental models of metastasis. In highly metastatic subclones isolated from human-derived cancer cell lines, the selection of pre-existing genetic mutations, such as *KRAS*^*G12D*^ and *BRAF*^*G46V*^, was associated with increased metastatic competence, suggesting no additional mutations were required.^52^ In a genetically engineered mouse model of CRC, mice bearing mutations in four genes commonly found in primary human cancers (*Apc*^*fl/fl*^, *Kras*^*LSL-G12D*^, *Tgfbr2*^*fl/fl*^ and *Trp53*^*fl/fl*^) formed metastases.^53^ Interestingly, no metastases were seen in mice that lacked the full set of four mutations.^53^ Consistent with this observation, exome sequencing data from CRC patients with brain or liver metastases revealed that various combinations of early driver genes collectively disrupting key signalling pathways (WNT, TP53, TGFB, EGFR and cellular adhesion) were significantly enriched in metastases.^34^ These studies suggest that although no specific mutations underlying metastasis seem to exist, specific combinations of mutations in primary tumours, possibly acquired in a particular order, can lead to enhanced metastatic competence. In addition, the existence of genes whose overexpression inhibits metastasis but not primary tumour growth in experimental systems (i.e. functionally defined metastasis suppressors) suggests that there are pathways specifically linked to metastasis, raising the possibility that further sequencing could potentially lead to the identification of metastasis-specific genetic driver lesions that have yet to be discovered.^54^

### Chromosomal aberrations and metastasis

The genetic feature most clearly associated with metastatic cancer is whole-genome doubling (WGD). Chromosomal instability (CIN) is a process resulting in chromosome abnormalities and aneuploidy, which correlate with poor prognosis and metastasis across multiple cancer types.^25,55–57^ Specifically, an increase in chromosome aberrations during tumour progression has been reported in renal, CRC, prostate, pancreatic and breast cancer in independent studies,^42,45,58–61^ as well as in mouse models of melanoma^62^ and pancreatic cancer.^63^ WGD has been found in up to 80% of metastases in certain types of cancer.^24^ Although WGD is commonly found in metastases, this varies depending on the cancer type and is not exclusive to metastasis as it has been observed in around 30% of primary tumours as well.^64^ Interestingly, cancer cells displaying WGD may be more tolerant of CIN in general, possibly facilitating further genetic evolution of metastases.^65^

One possible explanation for the association between CIN and metastasis is that CIN is a mechanism for the genetic amplification of oncogenic signalling, resulting in more aggressive cancer clones. In support of this notion, Priestley et al. found twice as many driver gene amplifications in samples with WGD events.^24^ In addition, multi-region profiling of primary and metastatic CRC found that chromothripsis (a mutational process in which chromosome shattering leads to complex rearrangements) and focal amplifications of *MYC* were features of metastatic tumours,^56^ while another study also identified *MYC* as well as *YAP1* amplifications in brain metastases from lung cancer patients.^47^ Gains in the number of oncogenic mutant *KRAS* alleles have been reported in pancreatic cancer metastasis.^45^ Another potential mechanism by which CIN can lead to metastasis has been proposed by Bakhoum et al.^66^ In this model, high CIN results in increased levels of cytosolic DNA, which, in turn, triggers the cGAS-STING cytosolic DNA sensing pathway and downstream activation of noncanonical nuclear factor (NF)-κB signalling, leading to epithelial–mesenchymal transition (EMT). Inhibition of NF-κB signalling was found to decrease metastasis in a human-derived cancer cell line model.^66^ Alternatively, the possibility also remains that CIN could be a consequence of tumour evolution rather than a functional driver of it. For instance, loss of p53 function is associated with poor prognosis and can lead to CIN;^67^ it is therefore possible that CIN is a passenger event that occurs as a consequence of a driver event such as p53 inactivation. Analyses of large cancer genome datasets have revealed that WGD is enriched in tumour types with extensive loss-of-heterozygosity (LOH), suggesting that WGD might be selected for during tumour evolution to mitigate deleterious somatic mutations and somatic copy number changes in regions of LOH.^68^

Despite a lack of comprehensive understanding of the mechanisms underlying the association between CIN and metastasis, the evidence linking these features is compelling and indicates that CIN could be a possible genetic means by which cancer clones acquire metastatic capabilities.

### Therapy- and immunity-induced selection and metastasis

The genetic evolution of metastatic tumours is shaped by tumour-extrinsic factors. The strong selective pressures induced by exposure to treatment result in acquired drug resistance mutations, parallel evolution and relapse. Drug-treated metastases have been reported to harbour private driver mutations unlike untreated metastases.^43^ Although resistance mutations can arise de novo,^69^ they also frequently pre-exist as minor subclones.^70^ In patients with breast cancer, around 20% of metastatic tumours develop mutations in the gene encoding oestrogen receptor 1 (*ESR1*) during endocrine therapy.^71^ Larger-scale reports have suggested that therapy-resistant metastatic breast cancer could also acquire new driver alterations in genes other than *ESR1*, such as those involved in the mitogen-activated protein kinase (MAPK) pathway.^72^ In another study of five patients, *ERBB2* mutations were acquired during endocrine therapy.^73^ *ESR1* and *TP53* driver mutations, as well as amplifications in *MDM4*, *FGFR1* and *CCND1*, were detected in patients relapsing after endocrine therapy, and mutations in SWI/SNF genes were found following taxane chemotherapy.^42^ In addition, parallel evolution of distinct *PTEN* mutations was detected in metastases following acquired resistance to an inhibitor of phosphatidylinositol 3-kinase α.^74^

In addition to therapy-induced mutations, pressures from the tumour microenvironment can provide an environment for positive selection and parallel evolution of metastasis-initiating cells. One such example comes from a longitudinal study of patients with metastatic CRC in which genetic data were consistent with parallel selection during metastasis evolution dependent on the strength and quality of the local immune response: metastatic clones could be traced back to immune-privileged clones that were capable of escaping the intrametastatic immune microenvironment, while the eliminated clones were immunoedited.^75^ Interestingly, LOH of HLA, which impairs the ability of the immune system to recognise tumour antigens, is associated with metastasis,^76^ and different metastases can be characterised by very different immune microenvironments even within a single patient.^77,78^ These observations highlight not only the continuous genetic evolution of metastases influenced by changing environmental pressures, but also the challenges of keeping up with the evolving metastases during therapy.

## Epigenetic origins of metastasis

As outlined in the previous section, specific metastasis-causing mutations have not been identified. Nevertheless, primary cancers with similar tumour-initiating mutations and other early mutations can eventually progress towards vastly different metastatic phenotypes, specific transcriptional programmes correlate with poor patient prognosis and metastasis in several, if not all, tumour types, and stable highly metastatic cancer clones with distinct gene expression programmes can be isolated from multiple different human- and mouse-derived experimental cancer models.^33,79^ Moreover, extensive experimental data support the idea that these gene expression programmes contain functionally important mediators of various metastatic phenotypes.^80^ So, specific transcriptional programmes appear to underlie the development of metastases, but they don’t seem to be induced by specific metastasis-associated mutations. This presents a central problem: how do the metastatic transcriptional traits arise?

**Fig. 2:**
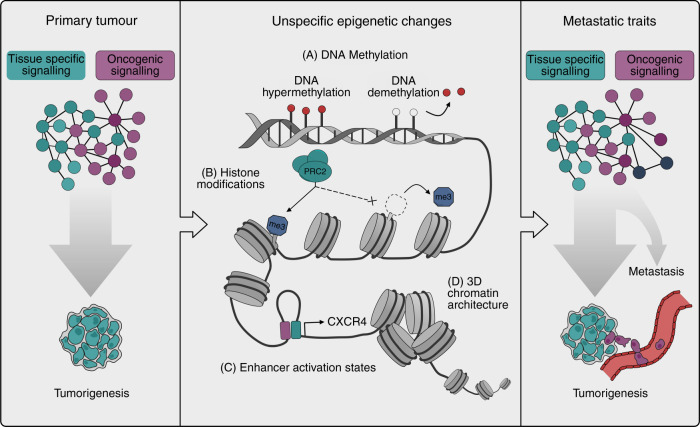
Epigenetic mechanisms alter oncogenic signalling in support of metastasis. Genetically activated pathways and tissue-specific cellular programmes drive oncogenic signalling in primary tumours (left). The phenotypic output of these pathways can change via several epigenetic mechanisms ranging from alterations in DNA methylation (**a**), chromatin accessibility (**b**), histone modification states (**c**) and higher order chromatin conformation (**d**). As these epigenetic mechanisms are in principle unspecific, the actual phenotypes that emerge are dictated by the oncogenic programmes that are active in the cells. Some of these phenotypes will enhance oncogenic signalling and get selected for. The phenotypic output of the oncogenic pathways that drive primary tumour formation thus evolves via epigenetic alterations to support metastatic cancer phenotypes (right).

Various heritable nongenetic, i.e. epigenetic, mechanisms have been linked to cancer progression. For example, large-scale alterations in DNA methylation, chromatin accessibility, histone modifications and 3D chromatin conformation are typically observed in cancer and many such alterations also correlate with cancer prognosis and metastasis.^79^ Strong evidence links multiple different factors such as mutations in genes that encode for chromatin-associated proteins (e.g. members of the SWI/SNF chromatin remodelling complexes),^81^ oncometabolites^82^ or environmental factors such as hypoxia^83^ to altered chromatin in cancer. However, as most of these epigenetic mechanisms associated with cancer development and progression cause unspecific (i.e. not directly guided by the underlying DNA sequence) perturbations in chromatin states, it is unclear how they alone can result in the activation of specific metastatic transcriptional programmes. The role of widespread chromatin alterations in metastasis may therefore be to facilitate the phenotypic evolution of genetically activated oncogenic programmes, allowing them to aberrantly activate various developmental, regenerative and stem-cell pathways in contexts where these pathways increase metastatic fitness (Fig. 2). The general epigenetic mechanisms that underlie cancer progression have been reviewed elsewhere.^79^ Here we focus on the nature of the epigenetically encoded cellular programmes that facilitate metastasis and discuss their interaction with genetically activated oncogenic pathways.

### Developmental and regenerative origins of metastatic programmes

One possible origin of metastatic traits is the activation of normal developmental or regenerative programmes in the wrong context. For example, L1CAM is a neural cell adhesion molecule that mediates metastatic colonisation in lung, breast, colorectal and other cancer types.^84–86^ In normal intestine, L1CAM is only expressed when epithelial damage occurs. In CRC, L1CAM is dispensable for adenoma formation, but is activated as tumours progress, in response to the loss of epithelial integrity as a result of the loss of E-cadherin in cell–cell junctions by a mechanism that requires the removal of REST (a regulator of the Polycomb receptor complex 1—PRC1) from an L1CAM enhancer. The transcriptional programmes induced by L1CAM govern the interaction between metastatic cells and the stroma in distant organs and are important for metastasis growth.^86^ Similarly, the transcription factors RUNX2, which is essential for bone development and osteoblast differentiation,^87,88^ supports metastasis in osteosarcoma,^89^ nuclear factor 1 B‑type (NFIB) activates neuronal enhancer programmes in support of lung cancer metastasis,^90^ and FOXA1 supports pancreatic cancer metastasis through a transcriptional program similar to those normally active in embryonic foregut endoderm.^91^ Interestingly, BORIS, a germ-cell-specific paralogue of the zinc-finger-binding protein CTCF, is overexpressed in several cancers. In treatment-resistant neuroblastoma, BORIS-regulated alterations in enhancer–promoter interactions support advanced cancer phenotypes,^92^ suggesting a possibility that similar mechanisms could underlie the activation of other developmental programmes in cancer as well. Thus, programmes that normally support tissue development and regeneration are constitutively and aberrantly activated in cancer cells, leading to enhanced metastatic fitness. However, how the activation of these programmes is linked to the genetic aberrations that drive carcinogenesis remains in most contexts poorly understood.

**Fig. 3:**
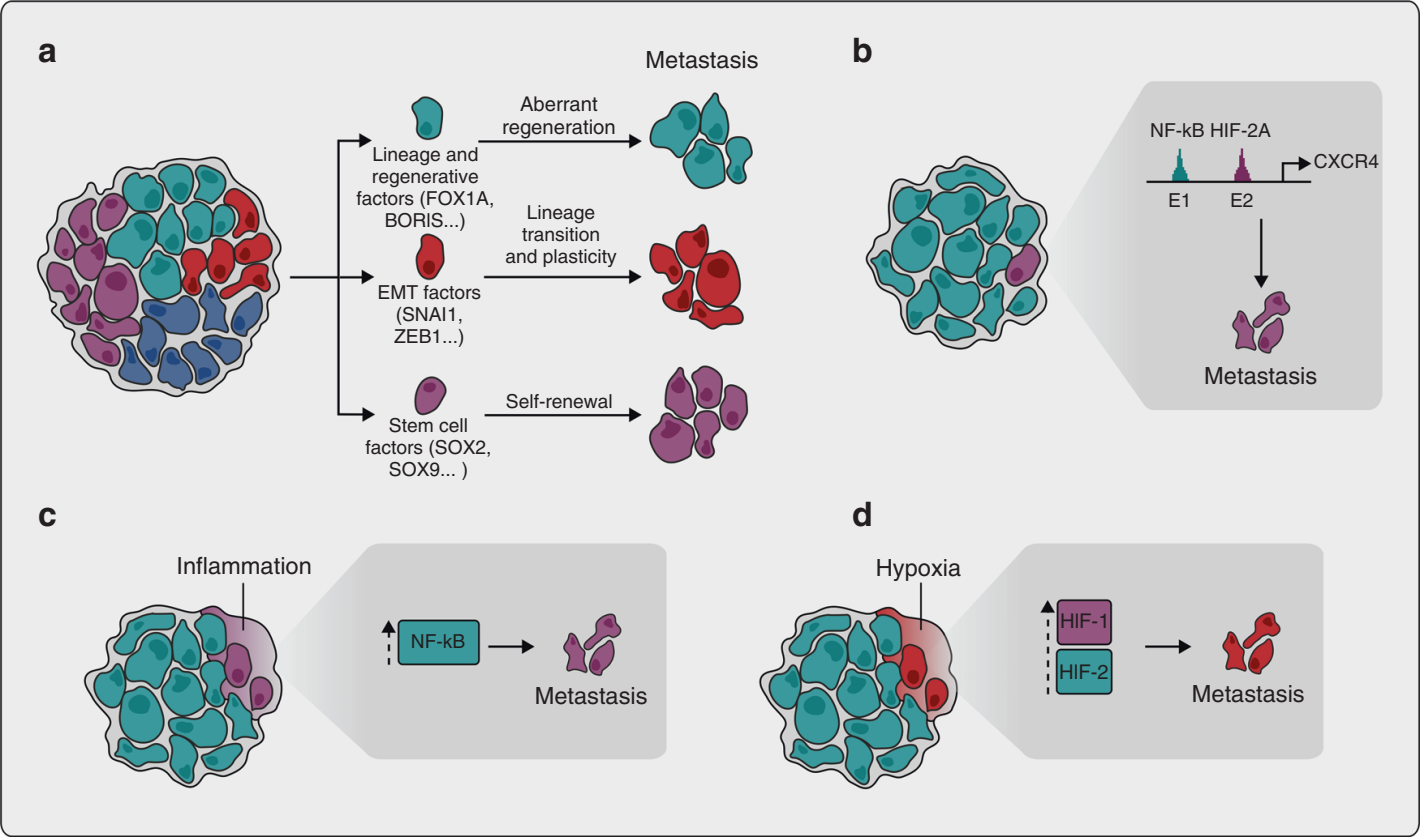
Epigenetic origins of metastatic transcriptional programmes. Aberrant activation of developmental and regenerative programmes, mediators of epithelial–mesenchymal transition (EMT), and stem-cell functions in cancer cells can lead to enhanced metastatic fitness (**a**). Genetic and epigenetic alterations resulting in optimisation of the phenotypic output of already activated oncogenic signalling pathways lead to the acquisition of metastatic traits. In kidney cancer, the output of the VHL–HIF-2A pathway is altered in metastatic clones through nuclear factor (NF)-κB-dependent enhancer co-option (**b**). Microenvironmental stimuli induce metastatic oncogenic signalling (**c**, **d**). The tumour microenvironment is characterised by persistent inflammation, inducing NF-κB activation and the expression of metastasis genes (**c**). Tumour hypoxia activates HIF1 and HIF2-dependent transcriptional programmes that have been linked to metastatic progression (**d**).

In addition to regulating lineage-specific developmental and regenerative programmes, factors that control lineage transitions during development can also support metastatic phenotypes. An obvious example is provided by the various mediators of EMT, which can establish cellular states with enhanced metastatic potential.^93^ Specific transcription factors, such as SNAI1, SNAI2, TWIST1 and ZEB1, orchestrate and coordinate the EMT programme. The activation of EMT factors has been widely studied and a remarkably complex picture has emerged with links to microenvironmental signals, oncogenic signalling pathways, epigenetic factors such as DNA methylation, metabolism and post-transcriptional regulation.^82,94–97^ Some, but not all, EMT transcription factors are important for metastasis in pancreatic,^98^ breast^99,100^ and skin cancer^101,102^ as well as other cancer types.^96,103^ Other EMT events, however, such as loss of E-cadherin expression, have been shown to inhibit metastasis;^104^ additional examples of context specificity of EMT factors as mediators of metastasis also exist.^105^ Hence, the role of EMT as a general phenomenon in metastasis, in contrast to the specific effects of the various EMT factors in different cancer contexts, still needs further clarification, but it is clear that several of these factors facilitate metastatic progression at least in some cancers (Fig. 3a).

### Homoeostatic stem-cell programmes as a source of metastatic fitness

In many cancers, tumour propagation depends on a subpopulation of cells with stem-like characteristics. Although some of the stemness programmes might be dependent on oncogenic pathways, others seem to be closely related to homoeostatic stem-cell programmes.^17,106^ An interesting example of the association between stem-cell programmes and cancer has been described in CRC, where an Lgr5-positive subpopulation with tumour- and metastasis-initiating capabilities has been identified.^107^ Lgr5 also marks normal intestinal stem cells, suggesting that the physiological intestinal stem-cell programmes might be conserved in cancer. Other studies have, however, highlighted the role of Lgr5-negative CRC cells in the initiation of metastasis,^108^ suggesting that the relationship between tumour subpopulations could be dynamic. Nevertheless, the frequent and CRC-specific mutations in the tumour suppressor adenomatous polyposis coli (APC) and its known function as an inhibitor of the Wnt signalling pathway that regulates Lgr5 expression provides one of the clearest examples of the close links between cancer driver mutations and homoeostatic stem-cell programmes as a critical determinant of tumour phenotypes, including metastasis-initiating potential. Another example comes from lung cancer, in which SOX2 and SOX9, two transcription factors associated with a stem-cell-like state in lung and breast cancer cells, support metastatic progression in experimental mouse models.^109,110^ Both SOX2 and SOX9 are also important for the survival of disseminated lung and breast cancer cells in secondary organs under immune surveillance, as well as metastatic outgrowth under permissive conditions.^110^ Hence, in parallel with developmental and regenerative pathways, various homoeostatic stem-cell programmes can be co-opted by cancer cells, and this can lead to enhanced metastatic fitness (Fig. 3a).

### Enhanced oncogenic signalling as a driver of metastasis

Instead of co-opting entire developmental, regenerative or homoeostatic programmes, another possible mechanism by which cancer clones can acquire metastatic traits is through optimising or fine-tuning the phenotypic output of already activated oncogenic signalling pathways at the transcriptional level. Such changes in transcriptional output can arise through different routes and depend on both genetic and epigenetic alterations. For example, in prostate cancer, mutations in FOXA1 lead to altered chromatin states that modulate hormone-dependent transcriptional signalling through the androgen receptor.^111^ Interestingly, a specific subtype of FOXA1 mutation with increased DNA affinity and chromatin-binding pattern is enriched in metastatic tumours and causes invasive phenotypes in tissue culture assays, possibly via a Wnt-dependent mechanism.^112^ Other experimental and clinical evidence also supports the role of the Wnt pathway in prostate cancer metastasis.^113^ Specific cancer driver mutations can thus directly modulate the epigenetic landscape of a cancer cell, consequently leading to a more aggressive metastatic phenotype.

The functional output of oncogenic signalling can also be altered by changes in chromatin landscapes without specific mutations. In oestrogen receptor (ER)-positive breast cancer, the YY1 transcription factor modulates ER signalling, which leads to disease progression;^114^ and in osteosarcoma, metastatic lesions show AP-1-dependent enhancer activation.^115^ In *VHL-*mutant renal cancer, the functional output of the tumour-initiating VHL–HIF2A pathway is altered in metastatic clones through NF-κB-dependent co-option of specific lymphoid enhancers upstream of the chemokine receptor *CXCR4*.^116,117^ In this context, both HIF2A and NF-κB operate through separate distal enhancers, the combined action of which is required for expression of CXCR4 and consequent enhanced metastatic competence. As HIF2A is stabilised in renal cancer by tumour-initiating mutations in the *VHL* tumour suppressor, these data provide detailed insight into the mechanisms of interaction between cancer driver mutations and epigenetic events, and how this leads to cancer progression. General support for aberrant enhancer activation states in association with cancer progression has emerged from pan-cancer analyses.^118,119^ Altered chromatin landscapes thus allow oncogenic pathways to activate distinct target genes in some cancer clones, which can lead to increased metastatic fitness (Fig. 3b).

### Microenvironmental cues as inducers of metastatic oncogenic signalling

Apart from their random acquisition of mutations, it is not obvious how cancer cells acquire the capability to constitutively activate various transcriptional programmes that remain transient under physiological conditions. Although some transcriptomic rewiring might be caused by general stress signalling associated with tumour growth,^120^ exposure to various microenvironmental factors, such as signalling molecules, stromal cells, metabolites or hypoxia could—in a premetastatic but already advanced cancer cell—induce stable transcriptional responses that eventually increase metastatic fitness. Accumulating experimental evidence is starting to support such a model. For example, persistent inflammation is able to induce NF-κB activation in renal cancer cells,^121^ which, on a background of genetically activated HIF2A signalling, can lead to the expression of mediators of metastasis.^116,121^ Enhanced inflammatory signalling through NF-κB can also modulate Wnt signalling in CRC, highlighting the general relevance of inflammation as a modulator of oncogenic signal output.^122^

In addition to inflammation, other environmental cues could, in the context of already activated oncogenic signalling, lead to stable activation of metastasis genes. For example, HIF1- and HIF2-dependent transcriptional programmes have been linked to metastatic progression in several tumour types,^123–126^ suggesting that activation of the hypoxia response in primary tumours might be important for metastatic tumour growth in the secondary organs. Some of these effects can be explained by constitutive activation of HIF1 and HIF2 in the absence of hypoxia, but low oxygen tension can also lead to stable changes in gene expression.^83,127,128^ Interestingly in lung cancer, antioxidant exposure can lead to the activation of BACH1, a transcription factor that, in turn, promotes a specific glycolysis-dependent metabolic programme important for metastatic growth.^129,130^ This suggests that the specific metabolic milieu of cancer cells could lead to stable alterations in their transcriptomes, consequently enhancing metastasis. In line with this, extracellular fatty acids can enhance oral carcinoma metastasis.^131^ Collectively, these data point to an important role for the tumour microenvironment in the stable epigenetic reprogramming of cancer cell transcriptomes, which can lead to enhanced metastasis (Fig. 3c).

## Conclusions

Almost two decades ago, in the absence of present-day insight from the large-scale genomic analysis of metastasis, Bernards and Weinberg proposed that metastatic progression is driven by the same oncogenic mutations as primary tumours.^132^ This prediction has since then been largely validated by extensive genetic data. However, it is also clear that the phenotypic output of oncogenic mutations is different in different cancer clones, and that these differences constitute important determinants of metastatic fitness. This variability in oncogenic output can be largely ascribed to various epigenetic alterations that shape the transcriptomes of cancer cells.^79^ However, while specific examples of these mechanisms are being unravelled by detailed functional analysis in different contexts, how cancer mutations interact with various developmental, homoeostatic and regenerative programmes in support of cancer progression and metastasis is still poorly understood. A striking result from cancer genome studies is that mutational patterns even in metastatic tumours remain strongly tissue specific.^24,81^ This suggests that in order to understand the origins of metastatic cancer traits, a comprehensive understanding of how physiological and oncogenic programmes co-operate at different stages of cancer progression must be sought. Such understanding should also result in the identification of genetic and epigenetic biomarkers for early detection of metastasis. Current efforts on multiple fronts are working towards this goal.

## Data Availability

Not applicable.
